# Investigating the Relationship between Perceived Meal Colour Variety and Food Intake across Meal Types in a Smartphone-Based Ecological Momentary Assessment

**DOI:** 10.3390/nu13030755

**Published:** 2021-02-26

**Authors:** Laura M. König, Julia E. Koller, Karoline Villinger, Deborah R. Wahl, Katrin Ziesemer, Harald T. Schupp, Britta Renner

**Affiliations:** 1 Department of Psychology, University of Konstanz, P.O. Box 47, 78457 Konstanz, Germany; julia.koller@uni-konstanz.de (J.E.K.); karoline.villinger@uni-konstanz.de (K.V.); deborah.wahl@uni-konstanz.de (D.R.W.); katrin.ziesemer@uni-konstanz.de (K.Z.); harald.schupp@uni-konstanz.de (H.T.S.); britta.renner@uni-konstanz.de (B.R.); 2 Faculty of Life Sciences: Food, Nutrition and Health, Campus Kulmbach, University of Bayreuth, 95326 Kulmbach, Germany; 3 Centre for the Advanced Study of Collective Behaviour, University of Konstanz, 78464 Konstanz, Germany

**Keywords:** eating behaviour, food colours, vegetables, snacks, ecological momentary assessment

## Abstract

Although most people are aware of the health benefits of consuming sufficient amounts of fruit and vegetables, many do not adhere to current dietary recommendations. Recent studies have suggested meal colour variety as an intuitive cue for healthy and enjoyable lunch meal choices. The present study extends this research by testing the “colourful = healthy” association across meal types. Using smartphone-based Ecological Momentary Assessment, 110 participants recorded 2818 eating occasions over a period of eight days. For each eating occasion, a picture, a short written description of the meal, the meal type (breakfast, lunch, afternoon tea, dinner, snack) and the perceived meal colour variety were recorded. Foods were classified into seven food groups based on the pictures and descriptions. Data were analysed using multilevel modelling. For all meal types except afternoon tea which did not include vegetables, perceived that meal colour variety was positively related to vegetable consumption (*b*s ≥ 0.001, *t*s ≥ 3.27, *p*s ≤ 0.002, quasi-R^2^s ≥ 0.06). Moreover, perceived meal colour variety was negatively associated with sweets consumption for breakfast, dinner and snacks (*b*s ≤ −0.001, *t*s ≤ −2.82, *p*s ≤ 0.006, quasi-R^2^s ≥ 0.01). The “colourful = healthy” association can be generalized across meal types and thus may be a promising strategy to promote a healthier diet.

## 1. Introduction

Consuming fruit and vegetables is beneficial for health. For instance, regularly consuming fruit and vegetables is associated with reduced risk for several chronic diseases including cardiovascular diseases and overall mortality [1,2,3]. Moreover, fruit and vegetable consumption may also be beneficial for mental health and well-being, e.g., by reducing psychological distress (e.g., [4,5,6]). Accordingly, dietary guidelines provided by scientific societies and government agencies recommend to consume plenty of fruit and vegetables (e.g., [7,8]).

Although most people are aware of the recommendations and the health benefits of consuming fruit and vegetables [9], adherence to the recommendations is generally low [10,11]. For instance, in Germany, only 13% of adults consume the recommended amount of vegetables, and less than 41% meet recommendations for daily fruit intake [12]. Similar figures have been reported for many other countries including the US and Australia [13,14,15]. Several reasons for this have been discussed in the literature, with hedonic preferences such as disliking fruit and vegetables being among important barriers to fruit and vegetable consumption [16,17,18]. Hedonic aspects of food consumption might be an especially important trigger for food choice since they are related to increased brain activity in reward areas [19], and because they highlight the immediate benefits of eating, compared to long-term benefits such as improved health [20]. Facilitating hedonic perceptions may therefore be an avenue for promoting healthier food choices, including the consumption of more fruit and vegetables [4,21].

Accordingly, recent research has turned to emphasising sensory and hedonic aspects when promoting healthy eating (e.g., [22,23]). For instance, in a series of laboratory and field studies, König and Renner [24,25] tested a “colourful = healthy” association, showing that more colourful lunch meals contained a larger proportion of vegetables and that vegetable consumption could be increased by prompting participants to eat colourful lunch meals. Moreover, participants stated that colourful meals were especially tasty and enjoyable to eat. Using meal colours to prompt healthier food choices is thus a promising strategy for health promotion.

So far, testing of the “colourful = healthy” association was limited to lunch meals. Because similar foods are consumed for lunch and dinner [26] it could be hypothesised that eating colourful meals also increases the proportion of vegetables in dinners. However, different foods may be consumed for breakfast or as snacks, such as colourful breakfast cereals or candy, which may alter the meal’s colour palette and subsequently the success of the intervention. The present study therefore investigated whether the “colourful = healthy” association also translates to other meal types. Specifically, it aimed to test whether perceived meal colour variety was positively associated with the consumption of fruit and vegetables across meal types. Furthermore, it was investigated whether this relationship was also evident for sugary extras, particularly for breakfast and snacks. An eight-day smartphone-based Ecological Momentary Assessment was used to capture food intake and perception of meal colour variety in daily life (c.f. [24]), and to allow for the analysis of within-person effects.

## 2. Materials and Methods

### 2.1. Sample

**Figure 1:**
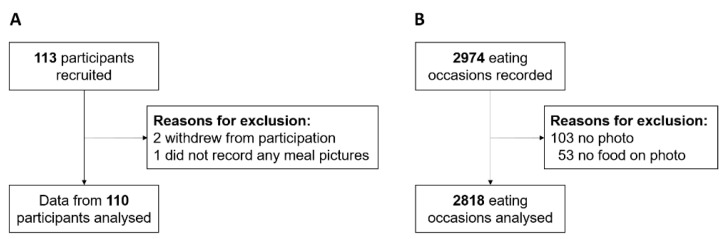
Flow diagram for (**A**) participants and (**B**) eating occasions.

One hundred thirteen participants were recruited through leaflets distributed at the University of Konstanz and through the department online study pool. All users in the study pool were eligible for participation unless they had defective colour vision. Two participants withdrew their participation and another participant did not record any meal pictures during the eight-day study period, which reduced the final sample to *n* = 110 (82.7% female; see Figure 1A for a flow diagram). The sample had a mean age of 22.02 ± 5.28 years and a mean body-mass index (BMI) of 21.89 ± 3.44. Most participants (96.4%) were students, of which the majority studied Psychology (69.8%). No participant reported defective colour vision (e.g., red green colour deficiency), thus no participant was excluded. Participants received 1.5 h of course credit or 15 € as compensation.

Across the eight-day study period, participants recorded 2974 eating occasions. Of these, 103 records were incomplete, i.e., did not include a food picture and the rating of meal colour variety, and 53 of the pictures that were recorded did not display any food. Thus, 2818 records of eating occasions were analysed (see Figure 1B for a flow diagram).

### 2.2. Procedure

The study was approved by the University of Konstanz ethics committee and carried out in accordance with the Declaration of Helsinki and the guidelines of the German Psychological Society. Participants were invited to the laboratory for introductory sessions and gave written informed consent. They then either installed the study app (movisensXS, version 1.1.1, available on Google Play for Android) on their own smartphone (*n* = 52) or received a study smartphone (*n* = 58; ASUS Padfone Infinity or Samsung Galaxy J5) with the app preinstalled. Subsequently, they filled in a pre-study questionnaire assessing demographics and eating behaviours. For the following eight days, participants were asked to record all meals and snacks, but not drinks, in the moment of consumption. This in-the-moment recording of an eating occasion included specifying the meal type, taking a picture of the meal, providing a short written description and rating the meal colour variety. If necessary, participants could also record leftovers by taking a picture. In addition, participants were able to indicate whether they had skipped a meal or snack and could record eating occasions later. To facilitate reporting, participants set times for two reminders that were sent in the morning and in the evening. Immediately after the eight-day recording period and four weeks later, participants were asked to fill in post-study questionnaires. The study was conducted as part of the project SMARTACT (uni-konstanz.de/smartact), funded by the German Ministry of Education and Research (Grant 01EL1820A). The present publication focuses on the food intake and the meal colour variety that were recorded in-the-moment. The remaining data are presented elsewhere [27,28].

### 2.3. Materials and Measures

#### 2.3.1. Food Intake

**Figure 2:**
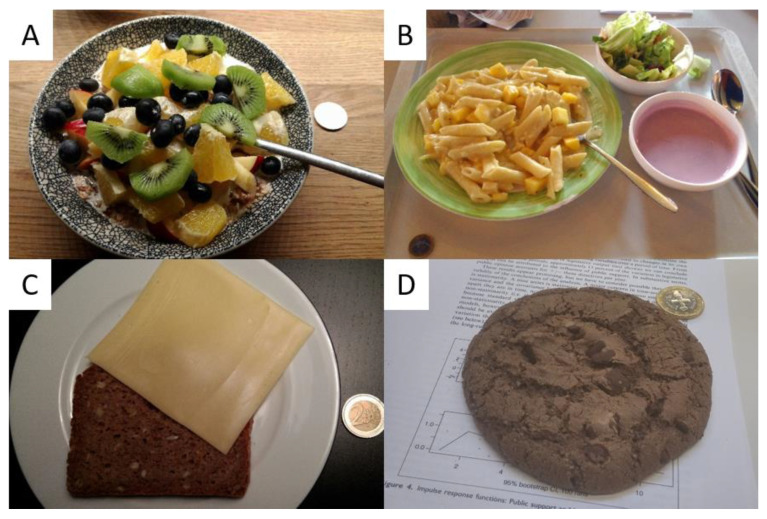
Example food pictures taken by participants. (**A**) Breakfast: 1.5 portions fruit (46%), 1 portion grains and starches (31%), 0.75 portions dairy (23%); (**B**) Lunch: 1 portion vegetables (17%), 1.5 portions grains and starches (25%), 2.5 portions dairy (42%), 1 portion fats (17%); (**C**) Dinner: 1 portion grains and starches (50%), 1 portion dairy (50%); (**D**) Snack: 1 portion sugary extras (100%).

Trained research staff coded food intake from the participant provided food pictures and meal descriptions according to a coding manual. In a first step, all foods were assigned to one of 16 categories of the German Nutrient Database (‘Bundeslebensmittelschlüssel’, BLS; Max Rubner Institut) and serving sizes were determined using a hand measure estimation aid that was based on German dietary guidelines [29]. In a second step, categories were reduced to seven food groups (vegetables, fruit, grains and starches, animal and other protein sources (i.e., ‘protein’), dairy, fats and oils, and desserts and other sugary foods (i.e., ‘sugary extras’)) to ensure comparability to previous research [24,25] and mirror German dietary guidelines [30]. For instance, the two BLS categories cereals and bread were grouped into one category labelled grains and starches. Interrater agreement and consistency were determined using two-way random single-measure Intraclass-Correlations (ICC_agreement_ [0.50; 0.92], ICC_consistency_ [0.51; 0.92], see also Table S1 in the supplementary material; [31,32,33,34]). As outlined by Cicchetti [34], an interrater agreement of 0.50 is considered fair, while an interrater agreement of 0.92 is considered excellent. As in König and Renner [24,25], a final intake score was computed per food category by dividing the portions served per category by the total amount of portions per meal, thus representing the proportion of the given category in the whole meal. Four examples are shown in Figure 2A–D.

#### 2.3.2. Meal Type

Participants classified the eating occasion into one of five categories: (1) breakfast, (2) lunch, (3) afternoon tea (i.e., traditional German afternoon meal for which cake and coffee or tea is served), (4) dinner, (5) snack.

#### 2.3.3. Perceived Meal Colour Variety

In accordance with previous research, meal colour variety was rated on a 100-point visual analogue scale ranging from ‘one colour’ to ‘many colours’ [24,25].

### 2.4. Statistical Analysis

Data were analysed using multilevel linear modelling [35] in R version 3.5.1 (Vienna, Austria) with the packages lme4 version 1.1-11 [36] and lmerTest 2.0-30 [37]. Models were computed for all eating occasions and separately for each meal type (breakfast, lunch, dinner, snack). As only 89 afternoon teas were recorded, models could not be computed separately for afternoon tea. For all analyses, individual meals defined Level 1, which were nested within participants (Level 2). Intra-class correlations (ICC) were computed using intercept only models. Relationships between perceived meal colour variety and intake of the seven food groups were analysed by entering perceived meal colour variety as a Level 1 predictor. Perceived meal colour variety was group-mean centred as recommended by Enders and Tofighi [38]. Both random slopes and random intercept models were then computed and compared using a deviance test [35]. If the deviance test was significant, differences between participants in the strength and/or direction of the relationship were assumed and the percentage of positive and negative slopes was computed [35]. In addition, quasi-R^2^ [39] was calculated as an estimate for the effect size, comparing the preferred model to the intercept only model.

## 3. Results

### 3.1. Perceived Meal Colour Variety

Average perceived meal colour variety was moderate; however, variation between eating occasions was substantial (38.47 ± 25.75, ICC = 0.16). Meal colour variety was highest for lunch (50.70 ± 24.85, ICC = 0.25), while it was lowest for snacks (24.50 ± 22.56, ICC = 0.25). Descriptive statistics are listed in Table 1.

### 3.2. Food Intake

Proportions of food groups in all meal types as well as descriptive statistics are listed in Table 2. Across all eating occasions, differential frequencies for the five different food groups emerged with grains and starches as the most prevalent group (63.31%) and fats and oils as the least prevalent group (12.38%). About one third of eating occasions included vegetables (39.03%) and dairy (34.88%) and about one quarter of occasions contained fruits (24.06%), protein (23.07%) or sugary extras (24.95%). Types of food consumed varied substantially across eating occasions, as indicated by ICCs ranging between 0.05 and 0.09. 

Within the five different meal types, differential distribution for the five food groups emerged (see Table 2). Grains and starches and vegetables were the two most frequently consumed food groups for lunches and dinners (>70 %). In addition, for breakfast, grains and starches were the most frequently consumed food group (80.63%) combined with dairy as the second most frequent group (48.30%). For snacks and in particular for afternoon teas, sugary extras were the most often consumed food group (snack: 40.81%; afternoon tea: 96.62%). ICCs were generally larger at the meal type level (e.g., ranging from 0.13 to 0.22 for breakfast) than at the eating occasion level, indicating that, within a meal type, more variance could be attributed to the participant level.

### 3.3. Relationships between Perceived Meal Colour Variety and Food Intake

For each food group, multilevel models were computed for all eating occasions and per meal type. A summary for the observed positive slopes can be found in Table 3. All model results are summarized in Tables S2–S6 in the online Supplementary Materials.

#### 3.3.1. Vegetables

A significant positive relationship was found between perceived meal colour variety and the proportion of vegetables consumed. When comparing the random slopes (*b* = 0.003, *t*(85.44) = 15.60, *p* < 0.001, quasi-R^2^ = 0.17) and random intercept models (*b* = 0.003, *t*(2705.93) = 21.18, *p* < 0.001, quasi-R^2^ = 0.14), the random slopes model assuming differences in the individual slopes was preferred (χ^2^(*df* = 2) = 28.82, *p* < 0.001). Therefore, it can be assumed that participants differed in the relationship between perceived meal colour variety and the proportion of vegetables consumed. However, 99% of slopes were positive, while only 1% of slopes were negative. Thus, for the vast majority of the sample, increased perceived meal colour variety was associated with a greater proportion of vegetables consumed.

This positive relationship was found consistently across meal types (breakfast: *b* = 0.001, *t*(74.29) = 4.83, *p* < 0.001, quasi-R^2^ = 0.10; lunch: *b* = 0.002, *t*(78.94) = 4.21, *p* < 0.001, quasi-R^2^ = 0.14; dinner: *b* = 0.003, *t*(85.99) = 5.41, *p* < 0.001, quasi-R^2^ = 0.11; snacks: *b* = 0.001, *t*(82.37) = 3.27, *p* = 0.002, quasi-R^2^ = 0.06). For all meal types, the random slopes model was preferred according to the deviance test (χ^2^s(*df* = 2) ≥ 6.74, *p*s ≤ 0.034; with 76% or more positive slopes within the different meal types (see Table 3).

#### 3.3.2. Fruit

A significant relationship emerged for the proportion of fruit consumed across eating occasions (*b* = −0.002, *t*(117.26) = −7.39, *p* < 0.001, quasi-R^2^ = 0.08). According to the deviance test, a random slopes model was preferred, indicating differences in the strength and/or direction of the relationship between participants (χ^2^(*df* = 2) = 77.65, *p* < 0.001). When analysing individual meal types, this negative relationship between meal colour variety and the proportion of fruit consumed was only found for snacks (*b* = −0.002, *t*(763.47) = −2.40, *p* = 0.016, quasi-R^2^ = 0.01). This time, the random intercept model was preferred (χ^2^(*df* = 2) = 0.13, *p* = 0.209), indicating that participants did not differ in the relationship between perceived meal colour variety and the proportion of fruit consumed as snacks.

#### 3.3.3. Grains and Starches

Across eating occasions, a significant positive relationship emerged for the proportion of grains and starches consumed (*b* = 0.001, *t*(84.94) = 3.36, *p* < 0.001, quasi-R^2^ = 0.04). The random slopes model was preferred (χ^2^(*df* = 2) = 30.69, *p* < 0.001). For the individual meal types, however, the direction of the relationship differed. While for snacks (*b* = 0.003, *t*(68.89) = 3.66, *p* < 0.001, quasi-R^2^ = 0.08) a positive relationship was also found, a negative relationship was found for breakfast (*b* = −0.002, *t*(57.14) = −2.99, *p* = 0.004, quasi-R^2^ = 0.08), lunch (*b* = −0.001, *t*(70.69) = −2.72, *p* = 0.008, quasi-R^2^ = 0.04), and dinner (*b* = −0.002, *t*(75.93) = −3.91, *p* < 0.001, quasi-R^2^ = 0.13). For all meal types, the random slopes model was preferred (χ^2^s(*df* = 2) ≥ 8.46, *p*s ≤ 0.015; breakfast: 68% of slopes negative; lunch: 76% of slopes negative; dinner: 73% of slopes negative; snacks: 75% of slopes positive).

#### 3.3.4. Protein

Across eating occasions, a significant positive relationship emerged between perceived meal colour variety and the proportion of protein (*b* = 0.002, *t*(147.57) = 10.02, *p* < 0.001, quasi-R^2^ = 0.06). The random slopes model was preferred according to the deviance test (χ^2^(*df* = 2) ≥ 30.36, *p* < 0.001; 96% of slopes positive). Significant positive relationships between perceived meal colour variety and the proportion of protein were also found for breakfast (*b* = 0.002, *t*(117.06) = 4.63, *p* < 0.001, quasi-R^2^ = 0.18), dinner (*b* = 0.001, *t*(660.74) = 2.28, *p* = 0.023, quasi-R^2^ = 0.01), and snacks (*b* = 0.001, *t*(31.93) = 3.46, *p* = 0.002, quasi-R^2^ = 0.11). Random slopes models were preferred for breakfast and snacks (χ^2^s(*df* = 2) ≥ 28.20, *p*s < 0.001), while the random intercept model was preferred for dinner (χ^2^(*df* = 2) = 0.084, *p* = 0.959). Thus, the strength and/or direction of relationships differed between participants for breakfast and snacks, but not for dinners.

#### 3.3.5. Dairy

For dairy, only the relationship between perceived meal colour variety and proportion of dairy consumed for lunch reached significance. This relationship was negative (*b* = −0.001, *t*(77.48) = −2.06, *p* = 0.043, quasi-R^2^ = 0.13) and the random slopes model was preferred (χ^2^(*df* = 2) = 26.18, *p* < 0.001; 62% of slopes negative).

#### 3.3.6. Fats and Oils

Similarly, for fats and oils, only the relationship between perceived meal colour variety and the proportion of fats and oils consumed for breakfast reached significance. This relationship was positive (*b* = 0.000, *t*(130.72) = 2.14, *p* = 0.034, quasi-R^2^ = 0.13) and the random slopes model was preferred (χ^2^(*df* = 2) = 30.68, *p* < 0.001).

#### 3.3.7. Sugary Extras

A significant negative relationship emerged for sugary extras (*b* = −0.004, *t*(135.99) = −11.97, *p* < 0.001, quasi-R^2^ = 0.10), for which the random slopes model was preferred (χ^2^(*df* = 2) = 39.78, *p* < 0.001; 98% of slopes negative). Significant negative relationships also emerged for the relationships between perceived meal colour variety and the proportion of sugary extras consumed for breakfast (*b* = −0.002, *t*(123.65) = −3.21, *p* = 0.002, quasi-R^2^ = 0.04), dinner (*b* = −0.001, *t*(127.93) = −2.82, *p* = 0.006, quasi-R^2^ = 0.22), and snacks (*b* = −0.003, *t*(764.32) = −2.96, *p* = 0.003, quasi-R^2^ = 0.01). For breakfast and dinner, the random slopes model was preferred (χ^2^s(*df* = 2) ≥ 13.40, *p*s ≤ 0.001), while for snacks, the random intercept model was preferred (χ^2^(*df* = 2) = 3.76, *p* = 0.153).

## 4. Discussion

Previous research suggests that increasing meal colour variety may be a feasible and effective strategy to promoting healthier eating [25]. The present study successfully replicated and extended previous work [24], showing that across meal types, more colourful meals contain a greater proportion of vegetables and a smaller proportion of sugary extras. Accordingly, eating colourful meals may be a strategy to improve the nutritional quality of diet independent of the meal type.

This notion is in line with previous research highlighting the importance of visual cues [40,41] and especially food colour on food choice and consumption [42,43,44]. Food colour may provide information about flavour identity [45], intensity of taste [46], food quality [47], or energy content [48]. Food colours may also contribute to meals being perceived as visually aesthetic, which might increase likelihood of consumption [49]. Finally, in the context of fruit and vegetable consumption, food colours are associated with phytonutrient content, which gives food their colouring and is also related to the food’s nutritional value and health benefits [50,51].

Although fruits are as colourful as vegetables, the present study and previous studies ([24]; [25], Study 3) found either non-significant or significant negative relationships between meal colour variety and the proportion of fruit consumed. This lack of beneficial effect may be explained by fruit being often consumed in one piece, while vegetables are often consumed as mixed salads, mixed cooked vegetables or stews. This may be due to practical reasons, such as a whole banana being easier to prepare and to eat than a fruit salad. Moreover, this may be influenced by the study sample which consisted mainly of students, who often live in single households or do not share their food when co-habiting. They thus may not be able to store larger amounts of different kinds of fruit. Study 1 and 2 in König and Renner [25] highlight the importance of availability in this regard: In these two studies, the prompt to eat a colourful meal was tested in a laboratory study, in which, amongst others, a selection of pre-cut fruit was offered to participants. In this setting, the effect of the prompt to eat colourful meals was even stronger for fruit than it was for vegetables. Accordingly, future studies should include participants who have the opportunity to share their food with others (e.g., in families) or be conducted in environments where pre-cut fruit is available (e.g., in buffet-style cafeteria settings) to further test the effects of meal colourfulness on fruit consumption in real-life settings.

In the present study, meal colour variety was negatively associated with the proportion of sugary extras consumed overall and for breakfast, dinner and snacks. This finding contrasts with earlier studies on the relationship between food colours and consumption of candy which found that multi-coloured candy was consumed in greater amounts than single coloured candy [52,53]. It is important to note that these studies focused on single types of candy, while the present study took many different kinds of sugary extras consumed in real life into account. While candy is often colourful, many other sweets including chocolate, cakes and most types of sugary breakfast cereal are not, which might explain why negative effects were found for the broader category of sugary extras.

Assessing multiple eating occasions per participant allowed for exploring within-person variability and comparing the direction of the relationship between participants. Although participants differed in the strength of the relationship between meal colour variety and vegetable consumption, the direction of effects was remarkably similar. This indicates that prompting the consumption of colourful meals could be a generic approach to promoting healthier eating. For sugary extras, however, more variety in the relationships was observed for breakfast and dinner, for which 24% and 34% of the respective slopes were positive despite a general negative association. The relationship may thus be moderated, e.g., by culture [54,55], age [56,57] or preferences for certain kinds of sweets and their textures [58]. As the present study sample consisted of white Western students in a narrow age range, testing these moderators is open to further investigation. Similarly, future research needs to take individual preferences into account to explore their effects on the “colourful = healthy” association.

A strength of the present study is the comparably large sample of more than 100 participants who provided data on more than 2800 real-life eating occasions. However, participants were mainly female undergraduate students, and data were collected in a Western country. Generalization of the results to other populations and less affluent societies where costs are a more important barrier to fruit and vegetable consumption [59] may thus be limited. Furthermore, women typically consume more fruits and vegetables than men, thus the data on food consumption might not generalize to other samples comprising more male participants [60]. Moreover, the present study focused on recording meals and snacks to reduce the burden on participants. However, since beverages are a major source of energy (e.g., [61]), future studies investigating the “colourful = healthy” association should take beverages into account. Finally, the results presented are observational. Future studies need to test the “colourful = healthy” association across meal types in experimental and intervention studies to warrant conclusions about causality. Especially given the low proportion of breakfasts and snacks in which vegetables were consumed, the question arises whether a prompt to eat colourful would actually increase the consumption of vegetables, as they might not be considered typical breakfast or snack foods in Western diets [26] due to not tasting sweet and often being consumed cooked [62].

## 5. Conclusions

Although most people are aware of the health benefits of consuming sufficient amounts of fruit and vegetables, they often do not fulfil current recommendations. The present study also underlines the need for increasing fruit and vegetable consumption, as only 39% of meals contained vegetables, and 24% contained fruit. Extending previous research [24,25], the present study suggests that colourful meals have increased nutritional value because they contain a larger proportion of vegetables, and that this association is present in all meal types. This further supports the notion that prompting consumers to eat colourful meals may be an effective strategy for promoting healthier eating patterns. 

## Figures and Tables

**Table 1:** Descriptive statistics for meal colour variety.

Meal Type	M	SD	ICC
Across eating occasions	38.47	25.75	0.16
Breakfast	35.71	22.31	0.42
Lunch	50.70	24.85	0.25
Afternoon tea	25.83	17.58	0.47
Dinner	48.36	24.94	0.21
Snack	24.50	22.56	0.25

Note: M = mean; SD = standard deviation; ICC = Intraclass coefficient.

**Table 2:** Descriptive statistics for food groups consumed overall and per meal type.

Food Group	% of Meals	Proportion of Food Group in the Meal		ICC
		M	SD	
Across eating occasions (*n* = 2818)				
Vegetables	39.03	0.14	0.22	0.05
Fruit	24.06	0.14	0.31	0.05
Grains and starches	63.31	0.30	0.29	0.05
Protein	23.07	0.08	0.17	0.09
Dairy	34,88	0.12	0.21	0.06
Fats and oils	12.38	0.04	0.14	0.06
Sugary extras	24.95	0.18	0.35	0.05
Breakfast (*n* = 704)				
Vegetables	13.35	0.03	0.11	0.18
Fruit	39.49	0.16	0.26	0.22
Grains and starches	80.68	0.38	0.28	0.21
Protein	15.91	0.06	0.16	0.18
Dairy	48.30	0.19	0.23	0.14
Fats and oils	11.22	0.03	0.09	0.13
Sugary extras	31.68	0.15	0.28	0.16
Lunch (*n* = 566)				
Vegetables	77.39	0.28	0.24	0.15
Fruit	9.89	0.03	0.10	0.08
Grains and starches	86.40	0.38	0.25	0.19
Protein	37.63	0.12	0.18	0.13
Dairy	39.93	0.12	0.19	0.07
Fats and oils	18.20	0.06	0.14	0.06
Sugary extras	5.30	0.02	0.13	0.07
Afternoon tea (*n* = 89)				
Vegetables	0.00	0.00	-	-
Fruit	4.49	0.01	0.05	0.04
Grains and starches	2.25	0.02	0.12	0.80
Protein	1.12	<0.01	0.02	<0.01
Dairy	10.11	0.05	0.15	0.27
Fats and oils	0.00	0.00	-	-
Sugary extras	96.63	0.93	0.22	0.47
Dinner (*n* = 692)				
Vegetables	70.81	0.26	0.25	0.17
Fruit	8.67	0.03	0.13	0.24
Grains and starches	78.61	0.35	0.25	0.11
Protein	40.17	0.14	0.20	0.16
Dairy	43.79	0.13	0.19	0.14
Fats and oils	16.61	0.06	0.14	0.11
Sugary extras	7.37	0.05	0.18	0.10
Snack (*n* = 767)				
Vegetables	10.17	0.04	0.16	0.03
Fruit	36.51	0.32	0.45	0.10
Grains and starches	23.60	0.14	0.29	0.05
Protein	6.00	0.02	0.10	0.16
Dairy	13.69	0.07	0.20	0.12
Fats and oils	6.78	0.04	0.19	0.17
Sugary extras	40.81	0.35	0.46	0.09

Note: M = mean; SD = standard deviation; ICC = Intraclass coefficient.

**Table 3:** Percentages of positive slopes observed for the relation between perceived meal colour variety and food intake by meal type and food group. Empty cells indicate a non-significant relationship.

	Across Eating Occasions	Breakfast	Lunch	Dinner	Snacks
Vegetables	99% ^a^	83% ^a^	76% ^a^	85% ^a^	78% ^a^
Fruit	15% ^b^				0% ^c^
Grains and starches	96% ^a^	32% ^b^	24% ^b^	27% ^b^	75% ^a^
Protein	96% ^a^	76% ^a^		100% ^c^	70% ^a^
Dairy			38% ^b^		
Fats and oils		64% ^a^			
Sugary extras	2% ^b^	24% ^b^		34% ^b^	0% ^c^

^a^ significant positive relationship; ^b^ significant negative relationship; ^c^ random intercept model preferred.

## Data Availability

Data are available from the corresponding author upon reasonable request.

## Supplementary Materials

The following are available online at https://www.mdpi.com/2072-6643/13/3/755/s1, Table S1: Intraclass Correlations (ICC) for all food groups, Table S2: Results of the multilevel models to analyse the relationship between perceived meal colour variety and the consumption of seven food groups across meal types, Table S3: Results of the multilevel models to analyse the relationship between perceived meal colour variety and the consumption of seven food groups for breakfast, Table S4: Results of the multilevel models to analyse the relationship between perceived meal colour variety and the consumption of seven food groups for lunch, Table S5: Results of the multilevel models to analyse the relationship between perceived meal colour variety and the consumption of seven food groups for dinner, Table S6: Results of the multilevel models to analyse the relationship between perceived meal colour variety and the consumption of seven food groups as snacks.
