# Supplementary material for: Investigating the Relationship between Perceived Meal Colour Variety and Food Intake across Meal Types in a Smartphone-Based Ecological Momentary Assessment
Source: Nutrients. 2021 Feb 26;13(3):755. doi: 10.3390/nu13030755 (PMC7996926; doi:10.3390/nu13030755)
Supplement: Supplementary file 1 [file nutrients-13-00755-s001.pdf]

**Table S1.** Intraclass Correlations (ICC) for all food groups.

| Food group | Type | | Interpretation according to Cicchetti (1994) |
|---|---|---|---|
| | Agreement *ICC* | Consistency *ICC* | |
| Vegetables | .79 | .80 | Excellent |
| Fruit | .92 | .92 | Excellent |
| Grains and starches | .79 | .79 | Excellent |
| Protein | .85 | .85 | Excellent |
| Dairy | .66 | .67 | Good |
| Fats and oils | .50 | .51 | Fair |
| Sugary extras | .82 | .83 | Excellent |

**Table S2.** Results of the multilevel models to analyse the relationship between perceived meal colour variety and the consumption of seven food groups across meal types.

| Predictor | Random slopes model (fixed effects) | | | | | Random intercept model (fixed effects) | | | | |
|---|---|---|---|---|---|---|---|---|---|---|
| | *b* | *SE* | *t* | *df* | *p* | *b* | *SE* | *t* | *df* | *p* |
| *Model 1: proportion of vegetables* | | | | | | | | | | |
| Intercept | 0.141 | 0.006 | 22.51 | 103.05 | < .001 | 0.141 | 0.006 | 22.59 | 103.170 | < .001 |
| Perceived meal colour variety | 0.003 | 0.000 | 15.60 | 85.44 | < .001 | 0.003 | 0.000 | 21.18 | 2705.93 | < .001 |
| *Model 2: proportion of fruit* | | | | | | | | | | |
| Intercept | 0.137 | 0.009 | 15.24 | 101.14 | < .001 | 0.138 | 0.009 | 15.43 | 97.41 | < .001 |
| Perceived meal colour variety | -0.002 | 0.000 | -7.39 | 117.26 | < .001 | -0.003 | 0.000 | -11.08 | 2699.49 | < .001 |
| *Model 3: proportion of grains and starches* | | | | | | | | | | |
| Intercept | 0.299 | 0.008 | 36.70 | 102.68 | < .001 | 0.298 | 0.008 | 36.61 | 102.74 | < .001 |
| Perceived meal colour variety | 0.001 | 0.000 | 3.36 | 84.94 | < .001 | 0.001 | 0.000 | 5.60 | 2706.51 | < .001 |
| *Model 4: proportion of protein* | | | | | | | | | | |
| Intercept | 0.081 | 0.006 | 14.28 | 104.11 | < .001 | 0.081 | 0.006 | 14.36 | 103.46 | < .001 |
| Perceived meal colour variety | 0.002 | 0.000 | 10.02 | 147.57 | < .001 | 0.001 | 0.000 | 11.39 | 2704.20 | < .001 |
| *Model 5: proportion of dairy* | | | | | | | | | | |
| Intercept | 0.125 | 0.006 | 19.77 | 111.82 | < .001 | 0.125 | 0.006 | 19.77 | 112.08 | < .001 |
| Perceived meal colour variety | 0.000 | 0.000 | 1.16 | 79.99 | .250 | 0.000 | 0.000 | 1.50 | 2714.38 | .135 |
| *Model 6: proportion of fats and oils* | | | | | | | | | | |
| Intercept | 0.044 | 0.004 | 10.36 | 104.96 | < .001 | 0.044 | 0.004 | 10.40 | 105.08 | < .001 |
| Perceived meal colour variety | 0.000 | 0.000 | 1.67 | 84.54 | .099 | 0.000 | 0.000 | 2.90 | 2707.90 | .004 |
| *Model 7: proportion of sugary extras* | | | | | | | | | | |
| Intercept | 0.173 | 0.010 | 17.44 | 117.99 | < .001 | 0.173 | 0.010 | 17.58 | 116.47 | < .001 |
| Perceived meal colour variety | -0.004 | 0.000 | -11.97 | 135.99 | < .001 | -0.004 | 0.000 | -15.43 | 2719.09 | < .001 |

**Table S3.** Results of the multilevel models to analyse the relationship between perceived meal colour variety and the consumption of seven food groups for breakfast.

| Predictor | Random slopes model (fixed effects) | | | | | Random intercept model (fixed effects) | | | | |
|---|---|---|---|---|---|---|---|---|---|---|
| | *b* | *SE* | *t* | *df* | *p* | *b* | *SE* | *t* | *df* | *p* |
| *Model 1: proportion of vegetables* | | | | | | | | | | |
| Intercept | 0.036 | 0.006 | 6.24 | 89.05 | < .001 | 0.037 | 0.006 | 6.60 | 94.76 | < .001 |
| Perceived meal colour variety | 0.001 | 0.000 | 4.83 | 74.29 | < .001 | 0.001 | 0.000 | 5.98 | 701.11 | < .001 |
| *Model 2: proportion of fruit* | | | | | | | | | | |
| Intercept | 0.163 | 0.015 | 11.04 | 97.69 | < .001 | 0.162 | 0.015 | 10.87 | 101.33 | < .001 |
| Perceived meal colour variety | 0.000 | 0.000 | 0.60 | 79.71 | .553 | 0.000 | 0.001 | 0.53 | 696.49 | .599 |
| *Model 3: proportion of grains and starches* | | | | | | | | | | |
| Intercept | 0.371 | 0.014 | 26.01 | 96.61 | < .001 | 0.371 | 0.016 | 23.91 | 100.85 | < .001 |
| Perceived meal colour variety | -0.002 | 0.001 | -2.99 | 57.14 | .004 | -0.002 | 0.001 | -3.56 | 697.68 | < .001 |
| *Model 4: proportion of protein* | | | | | | | | | | |
| Intercept | 0.066 | 0.010 | 6.90 | 105.86 | < .001 | 0.064 | 0.009 | 7.32 | 113.72 | < .001 |
| Perceived meal colour variety | 0.002 | 0.000 | 4.63 | 117.06 | < .001 | 0.002 | 0.000 | 6.13 | 699.42 | < .001 |
| *Model 5: proportion of dairy* | | | | | | | | | | |
| Intercept | 0.190 | 0.012 | 16.48 | 103.37 | < .001 | 0.188 | 0.012 | 15.76 | 104.57 | < .001 |
| Perceived meal colour variety | -0.000 | 0.001 | -0.73 | 72.77 | .469 | -0.000 | 0.000 | -0.96 | 701.99 | .339 |
| *Model 6: proportion of fats and oils* | | | | | | | | | | |
| Intercept | 0.027 | 0.005 | 5.72 | 124.66 | < .001 | 0.026 | 0.004 | 5.95 | 139.28 | < .001 |
| Perceived meal colour variety | 0.000 | 0.000 | 2.14 | 130.72 | .034 | 0.001 | 0.000 | 2.82 | 701.78 | .005 |
| *Model 7: proportion of sugary extras* | | | | | | | | | | |
| Intercept | 0.148 | 0.014 | 10.75 | 96.41 | < .001 | 0.151 | 0.015 | 10.34 | 88.25 | < .001 |
| Perceived meal colour variety | -0.002 | 0.001 | -3.21 | 123.65 | .002 | -0.002 | 0.001 | -2.82 | 701.90 | .005 |

**Table S4.** Results of the multilevel models to analyse the relationship between perceived meal colour variety and the consumption of seven food groups for lunch.

| Predictor | Random slopes model (fixed effects) | | | | | Random intercept model (fixed effects) | | | | |
|---|---|---|---|---|---|---|---|---|---|---|
| | *b* | *SE* | *t* | *df* | *p* | *b* | *SE* | *t* | *df* | *p* |
| *Model 1: proportion of vegetables* | | | | | | | | | | |
| Intercept | 0.259 | 0.016 | 16.64 | 95.58 | < .001 | 0.258 | 0.014 | 18.86 | 127.20 | < .001 |
| Perceived meal colour variety | 0.002 | 0.001 | 4.21 | 78.94 | < .001 | 0.002 | 0.000 | 4.94 | 554.47 | < .001 |
| *Model 2: proportion of fruit* | | | | | | | | | | |
| Intercept | 0.026 | 0.007 | 3.73 | 90.60 | < .001 | 0.027 | 0.006 | 4.95 | 98.70 | < .001 |
| Perceived meal colour variety | -0.000 | 0.000 | -0.85 | 74.37 | .396 | -0.000 | 0.000 | -0.71 | 561.91 | .476 |
| *Model 3: proportion of grains and starches* | | | | | | | | | | |
| Intercept | 0.391 | 0.017 | 23.38 | 99.20 | < .001 | 0.391 | 0.015 | 25.46 | 127.61 | < .001 |
| Perceived meal colour variety | -0.001 | 0.000 | -2.72 | 70.69 | .008 | -0.001 | 0.000 | -2.71 | 548.37 | .007 |
| *Model 4: proportion of protein* | | | | | | | | | | |
| Intercept | 0.117 | 0.011 | 10.73 | 93.60 | < .001 | 0.116 | 0.010 | 11.35 | 135.65 | < .001 |
| Perceived meal colour variety | 0.000 | 0.000 | 0.03 | 278.22 | .973 | 0.000 | 0.000 | 0.19 | 557.53 | .853 |
| *Model 5: proportion of dairy* | | | | | | | | | | |
| Intercept | 0.128 | 0.012 | 10.69 | 97.58 | < .001 | 0.129 | 0.010 | 12.75 | 139.50 | < .001 |
| Perceived meal colour variety | -0.001 | 0.000 | -2.06 | 77.48 | .043 | -0.001 | 0.000 | -2.65 | 563.42 | .008 |
| *Model 6: proportion of fats and oils* | | | | | | | | | | |
| Intercept | 0.053 | 0.007 | 7.74 | 92.64 | < .001 | 0.053 | 0.007 | 7.41 | 143.73 | < .001 |
| Perceived meal colour variety | 0.000 | 0.000 | 1.33 | 72.84 | .187 | 0.000 | 0.000 | 1.41 | 563.69 | .158 |
| *Model 7: proportion of sugary extras* | | | | | | | | | | |
| Intercept | 0.025 | 0.006 | 3.92 | 110.11 | < .001 | 0.025 | 0.007 | 3.65 | 165.94 | < .001 |
| Perceived meal colour variety | -0.000 | 0.000 | -0.66 | 122.33 | .510 | -0.000 | 0.000 | -1.06 | 563.22 | .290 |

**Table S5.** Results of the multilevel models to analyse the relationship between perceived meal colour variety and the consumption of seven food groups for dinner.

| Predictor | Random slopes model (fixed effects) | | | | | Random intercept model (fixed effects) | | | | |
|---|---|---|---|---|---|---|---|---|---|---|
| | *b* | *SE* | *t* | *df* | *p* | *b* | *SE* | *t* | *df* | *p* |
| *Model 1: proportion of vegetables* | | | | | | | | | | |
| Intercept | 0.230 | 0.013 | 17.09 | 106.114 | < .001 | 0.232 | 0.013 | 17.43 | 123.57 | < .001 |
| Perceived meal colour variety | 0.003 | 0.000 | 5.41 | 85.99 | < .001 | 0.002 | 0.000 | 6.06 | 660.00 | < .001 |
| *Model 2: proportion of fruit* | | | | | | | | | | |
| Intercept | 0.034 | 0.008 | 4.32 | 108.31 | < .001 | 0.034 | 0.008 | 4.52 | 98.01 | < .001 |
| Perceived meal colour variety | -0.000 | 0.000 | -1.48 | 147.43 | .142 | -0.000 | 0.000 | -1.34 | 635.87 | .182 |
| *Model 3: proportion of grains and starches* | | | | | | | | | | |
| Intercept | 0.373 | 0.014 | 26.44 | 106.78 | < .001 | 0.367 | 0.013 | 28.17 | 124.66 | < .001 |
| Perceived meal colour variety | -0.002 | 0.001 | -3.91 | 75.93 | < .001 | -0.002 | 0.000 | -4.07 | 670.79 | < .001 |
| *Model 4: proportion of protein* | | | | | | | | | | |
| Intercept | 0.131 | 0.011 | 11.91 | 106.18 | < .001 | 0.131 | 0.011 | 11.81 | 122.93 | < .001 |
| Perceived meal colour variety | 0.001 | 0.000 | 2.31 | 589.23 | .021 | 0.001 | 0.000 | 2.28 | 660.74 | .023 |
| *Model 5: proportion of dairy* | | | | | | | | | | |
| Intercept | 0.128 | 0.011 | 11.95 | 105.51 | < .001 | 0.129 | 0.010 | 12.96 | 126.45 | < .001 |
| Perceived meal colour variety | -0.000 | 0.000 | -0.05 | 172.87 | .958 | -0.000 | 0.000 | -0.41 | 666.83 | .686 |
| *Model 6: proportion of fats and oils* | | | | | | | | | | |
| Intercept | 0.050 | 0.007 | 6.85 | 121.83 | < .001 | 0.050 | 0.007 | 7.08 | 135.31 | < .001 |
| Perceived meal colour variety | 0.000 | 0.000 | 1.69 | 67.13 | .096 | 0.000 | 0.000 | 1.71 | 673.67 | .087 |
| *Model 7: proportion of sugary extras* | | | | | | | | | | |
| Intercept | 0.053 | 0.011 | 4.75 | 112.66 | < .001 | 0.056 | 0.009 | 6.07 | 138.24 | < .001 |
| Perceived meal colour variety | -0.001 | 0.000 | -2.82 | 127.93 | .006 | -0.001 | 0.000 | -4.54 | 675.60 | < .001 |

**Table S6.** Results of the multilevel models to analyse the relationship between perceived meal colour variety and the consumption of seven food groups as snacks.

| Predictor | Random slopes model (fixed effects) | | | | | Random intercept model (fixed effects) | | | | |
|---|---|---|---|---|---|---|---|---|---|---|
| | *b* | *SE* | *t* | *df* | *p* | *b* | *SE* | *t* | *df* | *p* |
| *Model 1: proportion of vegetables* | | | | | | | | | | |
| Intercept | 0.057 | 0.009 | 6.56 | 76.17 | < .001 | 0.058 | 0.008 | 7.51 | 169.10 | < .001 |
| Perceived meal colour variety | 0.001 | 0.000 | 3.27 | 82.37 | .002 | 0.001 | 0.000 | 4.14 | 763.35 | < .001 |
| *Model 2: proportion of fruit* | | | | | | | | | | |
| Intercept | 0.293 | 0.025 | 11.96 | 67.02 | < .001 | 0.290 | 0.025 | 11.74 | 131.98 | < .001 |
| Perceived meal colour variety | -0.002 | 0.001 | -1.83 | 48.00 | .073 | -0.002 | 0.001 | -2.40 | 763.47 | .016 |
| *Model 3: proportion of grains and starches* | | | | | | | | | | |
| Intercept | 0.181 | 0.017 | 10.66 | 70.02 | < .001 | 0.176 | 0.015 | 12.07 | 132.71 | < .001 |
| Perceived meal colour variety | 0.003 | 0.001 | 3.66 | 68.89 | < .001 | 0.002 | 0.001 | 4.11 | 764.76 | < .001 |
| *Model 4: proportion of protein* | | | | | | | | | | |
| Intercept | 0.040 | 0.009 | 4.41 | 39.97 | < .001 | 0.036 | 0.006 | 5.83 | 88.10 | < .001 |
| Perceived meal colour variety | 0.001 | 0.000 | 3.46 | 31.93 | .002 | 0.001 | 0.000 | 4.23 | 752.30 | < .001 |
| *Model 5: proportion of dairy* | | | | | | | | | | |
| Intercept | 0.088 | 0.012 | 7.17 | 60.86 | < .001 | 0.087 | 0.012 | 7.55 | 105.59 | < .001 |
| Perceived meal colour variety | 0.001 | 0.000 | 1.69 | 31.52 | .101 | 0.001 | 0.000 | 1.72 | 760.32 | .086 |
| *Model 6: proportion of fats and oils* | | | | | | | | | | |
| Intercept | 0.041 | 0.010 | 4.08 | 110.26 | < .001 | 0.041 | 0.011 | 3.61 | 112.70 | < .001 |
| Perceived meal colour variety | -0.001 | 0.000 | -1.57 | 145.77 | .118 | -0.000 | 0.000 | -1.51 | 754.44 | .132 |
| *Model 7: proportion of sugary extras* | | | | | | | | | | |
| Intercept | 0.302 | 0.025 | 11.88 | 78.54 | < .001 | 0.315 | 0.024 | 12.87 | 138.16 | < .001 |
| Perceived meal colour variety | -0.003 | 0.001 | -3.18 | 51.07 | .002 | -0.002 | 0.001 | -2.96 | 764.32 | .003 |
